# Purification and Immobilization of Superoxide Dismutase Obtained from *Saccharomyces cerevisiae* TBRC657 on Bacterial Cellulose and Its Protective Effect against Oxidative Damage in Fibroblasts

**DOI:** 10.3390/biom13071156

**Published:** 2023-07-20

**Authors:** Phitsanu Pinmanee, Kamonwan Sompinit, Angkana Jantimaporn, Mattaka Khongkow, Dietmar Haltrich, Thidarat Nimchua, Prakit Sukyai

**Affiliations:** 1 Biotechnology of Biopolymers and Bioactive Compounds Special Research Unit, Department of Biotechnology, Faculty of Agro-Industry, Kasetsart University, Bangkok 10900, Thailand; phitsanu.pin@biotec.or.th; 2 Enzyme Technology Research Team, National Center of Genetic Engineering and Biotechnology (BIOTEC), Pathum Thani 12120, Thailand; kamonwan.sompinit@gmail.com (K.S.);; 3 Nanomedicine and Veterinary Research Team, National Center of Nanotechnology (NANOTEC), Pathum Thani 12120, Thailand; 4 Department for Food Science and Food Technology, University of Natural Resources and Life Sciences (BOKU), 1190 Vienna, Austria

**Keywords:** superoxide dismutase, enzyme purification, enzyme immobilization, bacterial cellulose, ROS elimination

## Abstract

Superoxide dismutase (SOD) is an essential enzyme that eliminates harmful reactive oxygen species (ROS) generating inside living cells. Due to its efficacities, SOD is widely applied in many applications. In this study, the purification of SOD produced from *Saccharomyces cerevisiae* TBRC657 was conducted to obtain the purified SOD that exhibited specific activity of 513.74 U/mg with a purification factor of 10.36-fold. The inhibitory test revealed that the purified SOD was classified as Mn-SOD with an estimated molecular weight of 25 kDa on SDS-PAGE. After investigating the biochemical characterization, the purified SOD exhibited optimal activity under conditions of pH 7.0 and 35 °C, which are suitable for various applications. The stability test showed that the purified SOD rapidly decreased in activity under high temperatures. To overcome this, SOD was successfully immobilized on bacterial cellulose (BC), resulting in enhanced stability under those conditions. The immobilized SOD was investigated for its ability to eliminate ROS in fibroblasts. The results indicated that the immobilized SOD released and retained its function to regulate the ROS level inside the cells. Thus, the immobilized SOD on BC could be a promising candidate for application in many industries that require antioxidant functionality under operating conditions.

## 1. Introduction

Superoxide dismutase (SOD) (EC 1.15.1.1) is an antioxidant enzyme that plays a crucial role in protecting cells against reactive oxygen species (ROS), particularly superoxide radicals (O_2_^−^), which are generated during stressful conditions [1]. SOD catalyzes the dismutation of superoxide radicals into hydrogen peroxide (H_2_O_2_) and molecular water (H_2_O), while hydrogen peroxide is subsequently converted into water by catalase or glutathione peroxidase, which is present in all organisms [2,3]. SOD effectively eliminates both endogenous free radicals produced during oxidative metabolism and exogenous oxidants generated by exposure to various stimuli [4]. Consequently, the function of SOD neutralizes numerous free radicals and protects cells from these harmful molecules. Furthermore, SOD is a metalloenzyme that requires a metal ion cofactor for its activity. SOD can be classified based on the metal ion binding at the active site, namely Cu/Zn-SOD, Mn-SOD, Fe-SOD, and Ni-SOD, each with distinct cellular locations and sensitivities to hydrogen peroxide and potassium cyanide [5,6,7]. With its potent antioxidant activity, SOD is widely applied in various industries, including cosmetics, food, agriculture, pharmaceuticals, and medicine, leading to a continuous expansion of the enzyme market [8,9,10,11]. However, despite its remarkable free radical-scavenging abilities, SOD is inherently labile and prone to degradation when exposed to an unstable environment [12]. Thus, maintaining its structural and functional stability poses significant challenges. Consequently, several techniques such as formulation, immobilization, or encapsulation have been widely employed to protect and enhance the stability of the enzyme under operational conditions [13,14].

Enzyme immobilization is a method that improves enzyme stability by preventing the target enzyme from undergoing structural denaturation caused by the external environment. This immobilization technique enhances the functional stability of the enzyme, allowing it to maintain its activity under various conditions [15,16]. There are several methods used for enzyme immobilization, including physical adsorption, covalent binding, cross-linking, entrapment, and membrane confinement. Each method is influenced by different factors that affect their performance. Immobilization can be performed by using inert and insoluble materials such as natural polymers, synthetic polymers, or inorganic materials [17]. Natural polysaccharides, like cellulose, gelatin, or collagen, as well as synthetic polymers like polyvinyl alcohol (PVA) and polycaprolactone (PCL), or supports such as Sepabeads, are commonly used as carriers. Inorganic materials, including silica, metal oxides, or clay tiles, can also serve as supports for immobilizing target enzymes [18,19,20,21,22,23]. Among these, bacterial cellulose (BC) has been widely introduced to immobilize enzymes and stabilize their structural and biological function.

BC is a naturally occurring biomaterial composed of linear chains of glucose residues, possessing unique characteristics such as unidirectional polarity, high purity, high porosity, biocompatibility, and strong mechanical and chemical properties. BC is produced by various microbial genera, including *Azotobacter*, *Gluconacetobacter* (formerly known as *Acetobacter*), and *Sarcina ventriculi*. Among these, *G. xylinus*, *G. hansenii*, and *G. pasteurianus* have been identified as excellent BC producers due to their high yield and purity [24,25]. Unlike plant cellulose, BC does not contain hemicellulose, lignin, or other components that require harsh chemical extraction methods [26]. The unique properties of BC make it suitable for immobilizing various substances, including chemicals, microbes, and enzymes, enabling controlled release and protection of the immobilized substances during operation [27]. For instance, chlorhexidine, a commonly used disinfectant in dental applications, has been successfully immobilized in modified and biodegradable BC, extending its release profile [28]. Furthermore, the immobilization of probiotic *Lactobacillus* on BC provided protection against gastric juices and bile salts solution, increasing the survival rate compared to free cells [29]. Enzyme immobilization on BC is a method which is increasingly utilized for several purposes. It allows enzymes to be retained within the support matrix, protecting them from extreme temperatures and pH changes. In a study by Dikshit and Kim [30], immobilized lipase on BC exhibited improved thermal and pH stability compared to the free enzyme. The structural properties of BC contribute to enhanced rigidity, providing protection against unfolding or denaturation under various conditions. The obtained results demonstrated the effectiveness of enzyme immobilization on BC in preserving the structural properties and biological function of the target enzyme. Consequently, the immobilization process provides protection to the immobilized substances in harsh environments, making them applicable in various industries including tissue engineering, dentistry, drug delivery, cosmetics, and environmental applications [31,32,33].

In this study, we conducted the production and purification of SOD obtained from *S. cerevisiae* TBRC657, which produces a high level of SOD. Our aim was to investigate the biochemical characteristics of the purified enzyme, including optimal conditions and the effect of pH and temperature on its stability. Subsequently, we immobilized the partially purified SOD on BC to assess its capacity and release profile. Furthermore, we compared the stability of the immobilized SOD and the free enzyme under various conditions. Additionally, we evaluated the antioxidant properties of the immobilized SOD by examining its effectiveness in eliminating ROS inside hydrogen peroxide-treated fibroblast cells. The obtained results suggested that immobilizing SOD on BC improved thermal stability of the enzyme compared to free SOD. Additionally, the immobilized enzyme still provided its antioxidant functionality, which could enhance the viability of fibroblasts under oxidative stress. Therefore, the immobilized SOD on BC could be a promising candidate for cosmetic, biomedical or other industries that require antioxidant functionality.

## 2. Materials and Methods

### 2.1. Microbial Isolates and Media Preparation

The strain *S. cerevisiae* TRBC657 and *G. xylinus* TISTR975 were deposited and can be accessed at Thailand Bioresources Research Center (TBRC) and Thailand Institute of Scientific and Technological Research (TISTR), Thailand, respectively. The culture media which used in this study were (1) YPD medium consisting of 1.0% (*w*/*v*) yeast extract, 2.0% (*w*/*v*) peptone and 2.0% (*w*/*v*) glucose; (2) SOD production medium consisting of 3.0% (*w*/*v*) yeast extract and 25.0% (*w*/*v*) molasses [34]; and (3) a coconut water-based medium for BC production consisting of 100.0 mL coconut water, 5.0 g sucrose and 2.5 g (NH_4_)_2_SO_4_ and the pH was adjusted to 4.5 with 5% (*v*/*v*) acetic acid [35].

### 2.2. Production of Crude SOD

The seed culture of *S. cerevisiae* TRBC657 was cultured by inoculating its single colony in a 20 mL YPD medium in a 50 mL Erlenmeyer flask and incubated at 35 °C, 250 rpm for 16 h. Then, the obtained culture was transferred in a 180 mL YPD medium in a 1 L Erlenmeyer flask, and cultured at 30 °C, 250 rpm for 16 h, which was further used as starter in a 5 L fermenter. After that, the obtained starter was transferred to 1.8 L of production medium in a BIOSTAT^®^ B Plus 5–L bioreactor (Sartorius, Göttingen, Germany). The cells were cultured at 35 °C with an agitation rate at 200 rpm and aeration rate at 0.5 vvm for 24 h. After that, the cells were harvested by centrifugation at 5000× *g* for 10 min and washed twice with a PBS buffer. The cells were resuspended with 50 mM sodium phosphate buffer (pH 7.5) with a ratio of wet cell weight to buffer volume of 1 g to 4 mL. Then, the cell suspension was disrupted by a high-pressure homogenizer with Microfluidics^TM^ M-110P (Microfluidics, Massachusetts, USA) at 15,000 PSI for 5 passages. The supernatant was harvested by centrifugation at 25,000× *g*, 4 °C for 1 h. The crude extract was measured for SOD activity (U/mL) at pH 7.0 and 35 °C with a SOD determination kit (Sigma-Aldrich, St. Louis, MO, USA). One unit (U) of enzyme was defined as the amount of required to inhibit the optical density of 440 nm of WST-1 formazan formation under assay conditions. The protein concentration (mg/mL) was determined by Bradford solution (Bio-Rad, Hercules, CA, USA) using bovine serum albumin (BSA) as the standard.

### 2.3. SOD Purification

The obtained SOD was purified by ammonium sulphate precipitation and followed by column chromatography. Firstly, enzyme precipitation was performed by adding ammonium sulphate salts ranging from 10% to 90% saturation at 4 °C for 1 h. The precipitates obtained from each fraction were recovered by centrifugation at 10,000× *g*, 4 °C for 10 min. Then, the protein pellet was redissolved with minimal volume of 50 mM sodium phosphate buffer (pH 7.5) and desalted by Amicon^®^ Ultra-15 Centrifugal Filter (Merck, Boston, MA, USA) with molecular weight cut-off at 10 kDa. The fraction yielding the highest SOD activity and purification factor were subjected to further purify by hydrophobic interaction chromatography (HIC).

The protein pellet obtained from 40–60% saturation ammonium sulfate was redissolved in 50 mM sodium phosphate buffer (pH 7.5), containing 2 M ammonium sulfate, 50 mM sodium chloride, 0.1 mM ETDA and 0.25 mM DTT. Then, the protein solution was applied to a Phenyl Sepharose^®^ 6 Fast Flow column with a volume of 80 mL which was equilibrated with the same buffer. Elution of the enzyme was performed by establishing a linear gradient with the buffer without ammonium sulfate at a flow rate of 5 mL/min. After that, the fraction containing SOD was desalted with 50 mM sodium phosphate buffer (pH 7.5) and continually purified by anion exchange chromatography (AEX). The protein solution was applied to a DEAE Sepharose^®^ Fast Flow column with a volume of 80 mL, which was equilibrated with the same buffer. Elution of the enzyme was performed by establishing a linear gradient with the buffer containing 1 M sodium chloride at a flow rate of 5 mL/min. Then, the fraction containing SOD was desalted with 50 mM sodium phosphate buffer (pH 7.5) and further purified by size exclusion chromatography (SEC). The protein solution was applied to a Superose^®^ 12 10/300 column with a volume of 1200 mL which was equilibrated with the same buffer. Elution of the enzyme was performed by establishing with the same buffer at a flow rate of 0.3 mL/min.

At each purification step, all fractions exhibiting a peak of 280 nm were collected. After desalting with the Sepharose^®^ G–25 column with a volume of 50 mL, the obtained protein solution was measured for SOD activity and protein concentration, as described in Section 2.2. The SOD activity and protein concentration were calculated for specific activity (U/mg), recovery yield (%) and purification factor obtained from each purification step. After that, SDS-PAGE was performed using Mini-PROTEAN^®^ TGX Stain-Free^TM^ precast gels with a gradient of 4–15% (Bio-Rad, Hercules, CA, USA). Protein samples were mixed with 2X Laemmli buffer (Bio-Rad, Hercules, CA, USA) and boiled for 5 min. The results were interpreted using a Gel Doc XRS + system and Image Lab (Bio-Rad, Hercules, CA, USA) program. All procedures were carried out according to the manufacturer’s recommendation.

### 2.4. pH and Temperature Optimization

The optimum pH of purified SOD was determined by measuring SOD activity at pH levels ranging from 4.0–11.0 using a universal pH buffer [36] at 35 °C. Meanwhile, the optimum temperature of purified SOD was determined by measuring SOD activity at temperatures ranging from 25–60 °C at pH 7.0. After that, the relative activity of the enzyme was calculated by comparing with its activity at optimal pH or temperature, which was set at 100%.

### 2.5. pH and Temperature Stability

The pH stability of purified SOD was determined by incubating the obtained SOD at pH levels ranging from 3.0–11.0 using the universal pH buffer at 4 °C for 1 h. Meanwhile, the temperature stability of purified SOD was determined by incubating the obtained SOD at temperatures ranging from 25–60 °C for 1 h. Then, the remaining activity of the enzyme was determined by comparing with its initial activity before incubation which was set at 100%.

### 2.6. Effect of Inhibitors

The effect of inhibitors was examined by incubating the purified SOD with 1.0 mM of hydrogen peroxide, potassium cyanide or sodium azide at 4 °C for 10 min. Then, the chemical-treated SODs were measured for their relative activities compared to untreated SOD.

### 2.7. Preparation of BC

In this study, a strain of *G. xylinus* TISTR975 was selected and used for BC production. A 10% (*v*/*v*) inoculum was added to the coconut water-based medium in 24-well plates. Microbial cultures were incubated at 30 °C for 7 days under static conditions. After incubation, the BC sheets that were produced on the surface of the medium were harvested and washed with distilled water to remove the residual medium and other impurities. Then, these BC sheets were boiled in 1% (*w*/*v*) NaOH solution for 2 h. After boiling, the BC sheets were purified by extensive washing in distilled water at room temperature until the pH of the water became neutral.

### 2.8. Immobilization of SOD on BC

The purified BCs were dried by lyophilization until completely evaporated. Then, the dried BCs were immersed in 10 mL of purified SOD solution with approximate total activity of 6500 units at 4 °C for 24 h. The SOD solution was sampled at 0.5, 1, 2, 4, 8, 12, 18 and 24 h of immersion and measured for its residual enzyme by assaying SOD activity. The immobilization capacity (%) of SOD on BC was calculated by ratio of residual total SOD activity and initial total SOD activity. Release profile of the 24 h immobilized SOD was investigated by immersing in PBS buffer at 4 °C for 24 h. The solution was sampled at 0.25, 0.50, 0.75, 1, 2, 4, 8, 12 and 24 h of immersion and the released enzyme was measured by assaying SOD activity. The immobilization capacity and release profile of SOD on BC, in terms of total enzyme activities, were plotted against time.

### 2.9. pH and Temperature Stability of Immobilized SOD

After purified SOD was immobilized on BC for 24 h, the pH stability of immobilized SOD was determined by incubating the immobilized SOD at pH levels ranging from 4.0–8.0 using the universal pH buffer at 4 °C for 1 h. Whilst the temperature stability of immobilized SOD was determined by incubating the immobilized SOD at temperatures ranging from 25–60 °C for 1 h. The treated immobilized-SODs were measured for their activities and their remaining activities were calculated compared to the untreated, immobilized SOD, which was set as 100%.

### 2.10. Protective Effect of Immobilized SOD against Oxidative Damage in Fibroblasts

The protective effect of immobilized SOD and free SOD on oxidative damage in fibroblast cells was investigated. Approximately 100,000 fibroblast cells were cultured in DMEM media (Thermo Fisher Scientific, Massachusetts, USA) supplemented with 4.5 mg/mL glucose and 10% (*v*/*v*) FBS in 24–well plates. Then, the fibroblast cells were maintained in an optimum condition at 37 °C in a humidified 5% CO_2_ incubator for 24 h. Then, fibroblast cells were treated with free SOD, empty BC or BC containing SOD, whilst the untreated cells were used as control. All reactions were continually incubated for 1 h. After BCs were removed, the fibroblast cells were supplemented with DMEM media and continually incubated for 24 h. Then, 100 µg/mL of hydrogen peroxide was individually added for 1 h. After that, the cell viability was measured by CellTiter-Glo^®^ Luminescent Cell Viability Assay (Promega, Madison, WI, USA). The luminescence signal was measured and calculated for the cell viability (%) compared to the untreated cells, which was set at 100%.

### 2.11. Statistical Analysis

All the data provided in the study is a mean of three replicates ± standard deviation, having performed a one-way ANOVA followed by Tukey’s Multiple Range Test using SPSS 11.5 at 5% level. Different superscripts within the same column and star signs within the same treatment indicated the significant differences (*p* < 0.05).

## 3. Results

### 3.1. Purification of SOD

**Figure 1:**
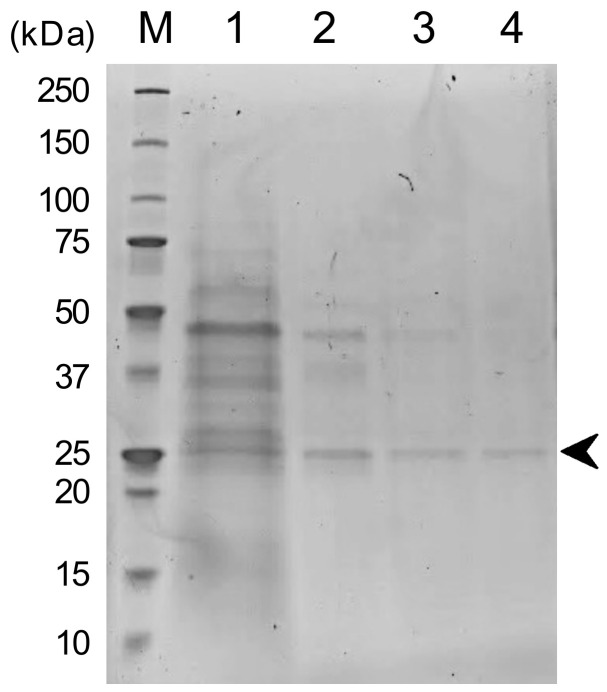
SDS-PAGE analysis of purified SOD obtained from *S. cerevisiae* TBRC657 (Lane M = molecular weight protein markers; lane 1 = partially purified SOD by salt precipitation; lane 2 = purified protein from HIC; lane 3 = purified protein from AEX; lane 4 = purified SOD from SEC).

As summarized in Table 1, SOD produced from *S. cerevisiae* TBRC657 was purified through the following steps: ammonium sulfate precipitation, HIC, AEX and SEC. The purified fraction of the resulting SOD displayed specific activity of 513.74 U/mg with a purification factor of 10.36-fold. In addition, a recovery yield was approximately 22.42% compared to the initial SOD concentration. Thus, SDS-PAGE analysis was performed to investigate the purity obtained from each step and predict its molecular weight of the purified SOD. According to Figure 1, each purification step successfully separated the impurities out of the protein of interest. Thus, lane 4 showed a single band of the purified SOD with an estimated molecular weight of 25 kDa, corresponding to the molecular weight of Mn-SOD [37,38].

### 3.2. Biochemical Characterization of Purified SOD

**Figure 2:**
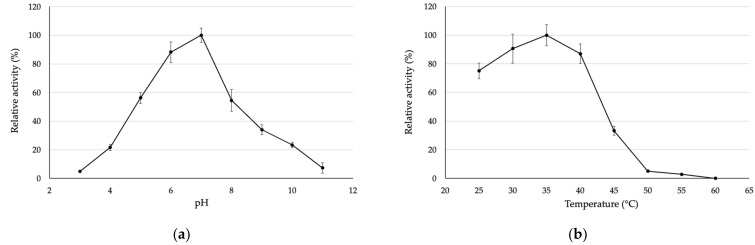
(**a**) pH and (**b**) temperature optimization of the purified SOD.

After the purified SOD was obtained, biochemical characterization was analyzed as follows. The optimum condition of the purified SOD was analyzed by monitoring the enzyme activity at different pH levels and temperatures. The results showed that an optimum pH of purified SOD was pH 7 and it still remained more than 80% of initial activity at pH 6 (Figure 2a). Furthermore, the purified SOD was examined its optimum temperature ranging from 25–60 °C (Figure 2b). The result exhibited that an optimum temperature of purified SOD was 35 °C. More than 70% of its relative activity was observed under assaying at 25–40 °C, whilst the SOD activity was rapidly decreased at 45 °C. Moreover, no enzyme activity was detected after the temperature of 50 °C was reached. The obtained results indicated that SOD is a labile enzyme that easily degrades and loses its biological activity when exposed to the environment.

**Figure 3:**
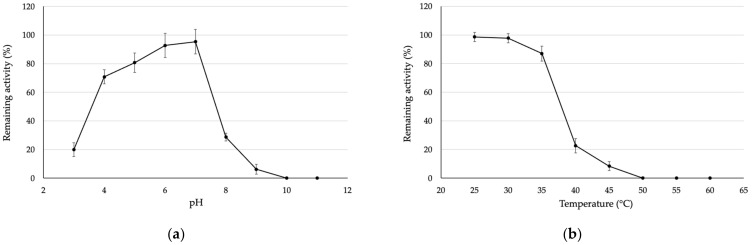
(**a**) pH and (**b**) temperature stability of the purified SOD after incubating at different pH levels and temperatures for 1 h.

Then, the stability of the purified SOD was investigated by incubating the purified SOD at different pH levels and temperatures for 1 h. After incubating the purified SOD at different pH levels ranging from 3–11 for 1 h (Figure 3a), the results showed that the purified SOD was stable at pH levels ranging from 4–7 with a 70% of relative activity compared to untreated SOD. On the other hand, the activity was rapidly decreased when incubating at the other conditions (Figure 3a). After that, the purified SOD was examined; its temperature stability ranged from 25–60 °C (Figure 3b) by incubating at different temperatures. As the result, the purified SOD was stable at temperatures ranging from 25–35 °C with remaining more than 90% of its activity, whilst the relative activity rapidly decreased at a temperature over 40 °C. The obtained results confirmed that SOD is a labile enzyme that easily degrades when the exposing environment is unsuitable [12].

Finally, the effect of inhibitors on purified SOD activity was also determined in order to categorize a type of the obtained SOD. As seen in in Table 2, the obtained SOD’s activity was not inhibited when incubating the enzyme with hydrogen peroxide and potassium cyanide, observing from the same level of SOD activity. On the other hand, the purified SOD lost its activity after incubating with sodium azide. Thus, this result indicated that the obtained SOD was categorized as Mn-SOD, which was not inhibited by hydrogen peroxide and potassium cyanide but partially inhibited by sodium azide.

### 3.3. Immobilization Capacity and Release Profile

**Figure 4:**
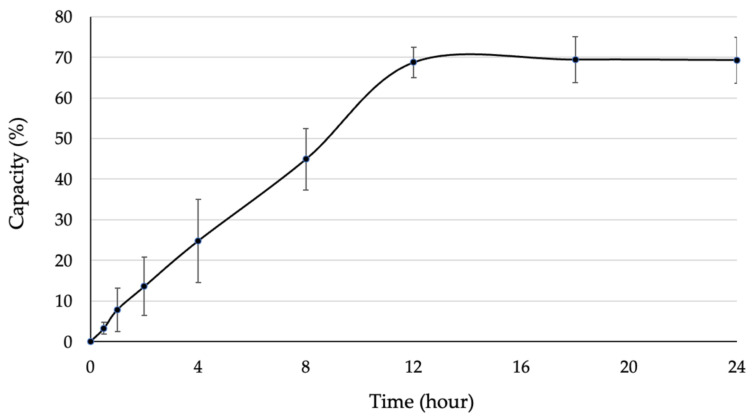
Immobilization capacity of SOD on lyophilized BC.

The obtained SOD was immobilized on bacterial cellulose produced by *G. xylinum,* in order to enhance its stability and investigate the protective effect of the immobilized SOD. Then, the immobilized capacity and release profile of SOD were examined to achieve the characteristic of immobilized SOD. When lyophilized BC was immersed with SOD solution, the enzyme was capable to access and immobilize on BC through its pores. After immersion, the remaining SOD in enzyme solution was measured, which represented the amount of SOD that did not immobilize on BC. As the result, the SOD was slightly accessed through the porous BC, resulting in an immobilization capacity of 70% at 0–12 h of immersion (Figure 4), whilst the immobilization capacity was constant at 70% since 12 h of immersion. Hence, immobilized SOD was entrapped inside the porous BC until reaching the equilibrium point. This result indicated that the BC reached a maximum capacity at 12 h and was ready for further analysis.

**Figure 5:**
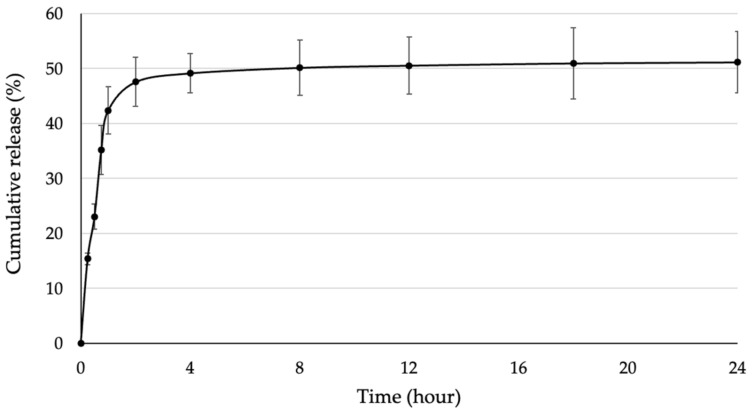
Release profile of immobilized SOD from BC.

The release profile of immobilized SOD (BC/SOD) was investigated to determine the treatment time and SOD dosage. The BC/SOD was immersed in a PBS buffer, and the released SOD was measured over time. The results revealed that approximately 50% of the initial amount of SOD was released from the BC. According to Figure 5, SOD was rapidly released within 1 h, after which the release rate remained relatively stable until 24 h. Therefore, the release profile of SOD from BC can be categorized into two stages based on the cumulative release. The first stage occurred within 1 h, with a cumulative release of 42.35% of the initial amount. This phenomenon is known as the burst effect, which represents a higher release rate at the beginning of the experiment. Following that, the second stage took place from 1 h until the end of the experiment, with a consistent release rate resulting in a cumulative release of 51.14% of the initial amount. Additionally, it was possible that a remaining SOD became aggregated within the porous structure of BC due to a higher concentration of the enzyme, leading to its entrapment within the BC material [39,40].

### 3.4. pH and Temperature Stability of Immobilized SOD

**Figure 6:**
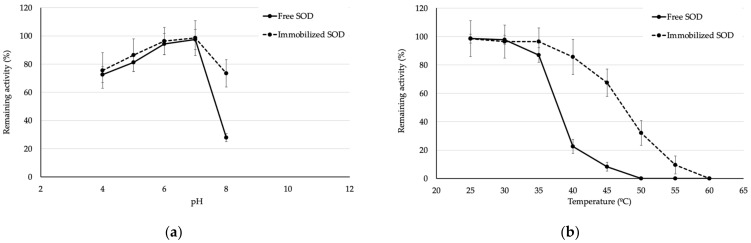
(**a**) pH and (**b**) temperature stability of the purified and immobilized SOD after incubating at different pH levels and temperatures for 1 h.

The stability of the immobilized SOD was investigated by incubating BC/SOD at different pH levels and temperatures for 1 h. Then, the treated BC/SODs were measured for their activities and were calculated as the remaining activity compared to the untreated BC/SOD. After incubating, the immobilized SOD was investigated at different pH levels, ranging from 4–8 for 1 h (Figure 6a); the results demonstrated that the immobilized SOD was stable at pH levels ranging from 4–8 with approximately 70% of remaining activity compared to the untreated BC/SOD, whilst the free SOD had lost 70% of initial activity when incubating at pH 8 for 1 h, due to its instability under this condition. After that, the immobilized SOD was examined its temperature stability by incubating at different temperatures ranging from 25–60 °C (Figure 6b). As the result, the immobilized SOD maintained more than 80% of its remaining activity at temperatures ranging from 25–40 °C. The remaining activity of the immobilized SOD gradually decreased from 40–45 °C and retained its activity more than 30% at 50 °C, whilst the free SOD rapidly decreased its activity from 40 °C (Figure 6b). The obtained results revealed that immobilization of SOD on BC enhanced its stability more than 40% after incubating at higher pH levels and temperatures compared to the free SOD, which typically degraded under those conditions. Therefore, the immobilization process using BC or other substances holds the potential to improve the stability of bioactive compounds under various conditions, which is a critical factor for the labile compounds [39,41].

### 3.5. Protective Effect of the Immobilized SOD against Oxidative Damage in Fibroblasts

**Figure 7:**
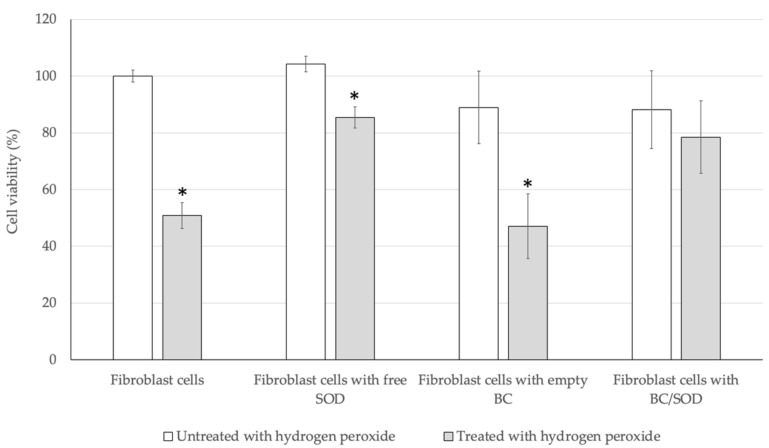
Cell viability of free SOD and immobilized SOD after treated with hydrogen peroxide for 1 h. Star signs (*) within the same treatment indicated the significant differences (*p* < 0.05) and values are presented as mean ± SD (*n* = 3).

Following this, the protective effect of the immobilized SOD (BC/SOD) on oxidative damage in fibroblast cells was investigated compared to the free SOD with the same amount of SOD released form BC/SOD and empty BC. An exogenous hydrogen peroxide generated a higher level of ROS inside the cells, followed by a programmed cell death phenomenon [42]. According to the results, those fibroblast cells that were directly treated with hydrogen peroxide shrunk, and did not attach to the others, which were the characteristics of programmed cell death with a viability of 50.85% compared to control. Whilst incubating fibroblast cells with the free SOD and treating with hydrogen peroxide, the cell viability test revealed that free SOD had potential to eliminate the ROS produced inside those cells with an approximate 35% increase in cell viability compared to the induced fibroblast cells (Figure 7). Interestingly, the numbers of fibroblast cells treated with empty BC or BC/SOD were significantly decreased by more than 10% of initial cells compared to control. Thus, BC was a material containing a bundle of porous where the cells might be located and attached on the BC. In addition, the empty BC did not contain any antioxidants involved in the ROS elimination, resulting in a decrease of cell viability on the same level as control. Meanwhile, the fibroblast cells that were incubated with BC/SOD and followed by hydrogen peroxide exhibited cell viability of 78.46%, which higher than the induced fibroblast cells (Figure 7). These results indicated that BC was non-toxic to fibroblast cells and facilitated the release of SOD, enabling the elimination of ROS generated within the fibroblast cells.

## 4. Discussion

SOD serves as a key component of the antioxidant defense system against oxidative stress in living organisms. It catalyzes the dismutation of superoxide radicals into harmless molecules. Moreover, SOD plays a significant role in maintaining free radical levels and preventing damages associated with oxidative stress that can be applied in various industries. In this study, we aimed to purify and examine the biochemical characterization of SOD obtained from *S. cerevisiae* TBRC657. Thus, the purification of SOD was carried out by ammonium sulfate precipitation, then hydrophobic interaction chromatography (HIC), anion-exchange chromatography (AEX) and size exclusion chromatography (SEC), respectively. According to these steps, the obtained enzyme showed specific activity of 513.74 U/mg with a 10.36-fold purification. Moreover, the purified SOD showed an estimated molecular weight of 25 kDa by SDS-PAGE and was insensitive to hydrogen peroxide and cyanide. These results indicated that the purified SOD was categorized as Mn-SOD, which can be found in the mitochondrial matrix [9].

The examination of biochemical characterization exhibited that the purified SOD had an optimal pH at 7.0. Typically, SOD’s activity is influenced by ionic strength and pH, which affects the charge of amino acid residues on the enzyme surface, especially lysine [43]. At a pH of 7.0, lysine carries a net charge very close to +1, leading to a positive charge on the surface of SOD. This positive charge facilitates superoxide radicals (O_2_^−^) to the active site of the enzyme, enabling efficient dismutation of the free radical. When the pH is shifted, the positive charge on the surface of SOD is reduced, resulting in a decrease of its activity. According to the optimal and stability tests under various temperatures, the purified SOD exhibited the highest activity at 35 °C. However, the purified SOD demonstrated a rapid decline in activity when exposed to high temperatures. The obtained results confirmed that SOD is categorized as a labile enzyme, which easily degrades when subjected to unstable conditions [12].

Thus, enhancing the structural and functional stability of the target enzyme is challenging. One commonly employed technique to protect the enzyme from such environments is enzyme immobilization. Besides the stability and functional efficiency of the target enzyme, immobilized enzymes can be reused multiple times, reducing the cost of enzyme production. They are compatible with continuous processes and can be applied in various fields, including biomedicine, environmental remediation, food processing, and cosmetics [17,44]. Inert polymers or inorganic materials are normally selected as support matrices with specific characteristics such as inertness, stability, regenerability and the ability to enhance enzyme stability. These materials are also non-toxic and biocompatible with the target enzymes [45]. Among those materials, bacterial cellulose (BC), which is an abundant natural polymer produced by *G. xylinus*, was used as carrier in this study. SOD was successfully immobilized on BC by entrapment, which involves trapping the enzyme in a porous structure through noncovalent bonds. This method offers several advantages such as a higher capacity for target enzyme loading and improved stability of the entrapped enzymes [46]. According to the results, the immobilized SOD demonstrated an enhancement in thermal stability when exposed to high temperatures, whilst the free SOD completely lost its activity. Thus, the support matrix of BC can protect the target enzyme from potential degradation or loss of activity, providing a stable and effective environment for enzyme function. The modification of BC, by the incorporation of functional groups or nanoparticles, may entrap the target enzyme through the specific bonding interaction, leading to effective immobilization and improved stability [47]. Moreover, the immobilized SOD was released from BC and eliminated ROS, which was generated inside hydrogen peroxide-treated fibroblasts, resulting in an increase in cell viability compared to cells without SOD treatment. The obtained results indicated that the immobilized SOD exhibited the stability improvement, particularly thermal stability, while still maintained its function as an antioxidant enzyme capable of eliminating ROS inside the cells. According to the results, the target bioactive molecules can be applied and immobilized on BC, resulting in a controlled release of these molecules from BC to the target location. The release of bioactive molecules allows them to function similarly to free molecules. Due to its functionality and thermal stability, the immobilized SOD can be applied in various applications such as biomedical, drug delivery, food or cosmetics. For biomedical applications, the immobilized SOD exhibits potent antioxidant properties with a slow release from the support, thereby preventing oxidative stress-related disorders. The immobilized enzyme can be used to treat conditions such as cardiovascular diseases, neurodegenerative disorders, and inflammation. Additionally, immobilization can provide a platform for SOD delivery systems, enabling the controlled release and targeted delivery of SOD at specific sites. For the food industry, immobilized SOD can be applied as a natural and stable antioxidant in food and beverages. Moreover, it is employed to inhibit oxidation and preserve the quality, flavor and nutritional value of products. Besides those, immobilized SOD can be utilized as an ideal ingredient for cosmetic and skincare products due to its stability under operating conditions. It helps combat skin aging by reducing the damage caused by free radicals and environmental factors such as UV radiation and pollution. Thus, further research in this area will provide great potential for developing strategies and products utilizing the immobilized SOD on BC or any supports, contributing to improved health, sustainability and biotechnological advancements.

## 5. Conclusions

SOD is an essential antioxidant enzyme found in all living organisms, which plays a crucial role in protecting against oxidative stress. Its primary function is to convert highly reactive superoxide molecules into less harmful oxygen and hydrogen peroxide. According to the results, the SOD produced from *S. cerevisiae* TBRC657 was successfully purified using the following steps: salt precipitation, hydrophobic interaction chromatography (HIC), anion exchange chromatography (AEX), and size exclusion chromatography (SEC), respectively. The inhibitory test revealed that the purified SOD belonged to the category of Mn-SOD, which is typically found in the mitochondrial matrix and is known to exhibit less stability compared to Cu/Zn-SOD. Despite the essential role of SOD as an antioxidant enzyme, it is considered as a labile enzyme with stability challenges. To overcome this limitation, various methods, especially immobilization techniques, have been applied to enhance the stability and preserve the functionality of the target enzyme. In this study, SOD was successfully immobilized on BC, resulting in an improvement in thermal stability against heat denaturation. Moreover, the immobilized SOD retained its ability to reduce superoxide radicals that were generated inside the ROS-induced fibroblast, resulting in an increase in cell viability. Thus, the immobilized SOD on BC could be a promising candidate for cosmetic applications that provide antioxidant protection and contributes to improved skin health and vitality. Additionally, the immobilized SOD can be applied in various industries, including biomedical, pharmaceutical, tissue engineering and other industries that require antioxidant functionality.

## Figures and Tables

**Table 1:** Purification factor of SOD obtained from each purification step.

Purification Step	Volume (mL)	Total Activity (U)	Total Protein Conc. (mg)	Specific Activity(U/mg)	Purification Factor
crude SOD	200.0	66,735.18	1351.20	49.55	1.00
salt precipitation	80.0	43,629.36	611.27	71.37	1.44
HIC	20.0	28,336.38	276.56	102.46	2.06
AEX	18.0	22,174.00	169.88	130.53	2.63
SEC	8.0	16,295.90	31.72	513.74	10.36

**Table 2:** Effect of inhibitors on SOD activity.

Inhibitors	Relative Activity (%)
Control	100.00 ± 8.92 ^a^
Hydrogen peroxide	97.92 ± 4.35 ^a^
Potassium cyanide	93.56 ± 6.35 ^a^
Sodium azide	67.06 ± 2.23 ^b^

Different superscripts within the same column indicated the significant differences (*p* < 0.05) and values are presented as mean ± SD (*n* = 3).

## Data Availability

The original contributions presented in this study are included in the article.
